# Risk Stratification and in Hospital Morality in Patients Presenting with Acute Coronary Syndrome (ACS) in Bahrain

**DOI:** 10.2174/1874192401812010007

**Published:** 2018-02-21

**Authors:** Taysir S Garadah, Khalid Bin Thani, Leena Sulibech, Ahmed A Jaradat, Mohamed E Al Alawi, Haytham Amin

**Affiliations:** 1 Salmaniya Medical Complex, Ministry of Health, Manama, Kingdom of Bahrain; 2 College of Medicine and Medical Sciences, Arabian Gulf University, Manama, Kingdom of Bahrain; 3 Bahrain Defense Force Hospital, Al Riffa, Kingdom of Bahrain

**Keywords:** Acute coronary syndromes, Bahrain, Risk factors, Mortality, GRACE score

## Abstract

**Background:**

Risk factors and short-term mortality in patients presented with Acute Coronary Syndrome (ACS) in Bahrain has not been evaluated before.

**Aim:**

In this prospective observational study, we aim to determine the clinical risk profiles of patients with ACS in Bahrain and describe the incidence, pattern of presentation and predictors of in-hospital clinical outcomes after admission.

**Methods:**

Patients with ACS were prospectively enrolled over a 12 month period. The rate of incidence of risk factors in patients was compared with 635 non-cardiac patient admissions that matched for age and gender. Multiple logistic regression analysis was used to predict poor outcomes in patients with ACS. The variables were ages >65 years, body mass index (BMI) >28 kg/m^2^, GRACE (Global Registry of Acute Coronary Events) score >170, history of diabetes mellitus (DM), systolic hypertension >180 mmHg, level of creatinine >160 μmol/l and Heart Rate (HR) on admission >90 bpm, serum troponin rise and ST segment elevation on the ECG.

**Results:**

Patients with ACS (n=635) were enrolled consecutively. Mean age was 61.3 ± 13.2 years, with 417 (65.6%) male. Mean age for patients with ST-segment elevation myocardial infarction (STEMI, n=156) compared with non-STEMI (NSTEMI, n=158) and unstable angina (UA, n=321) was 56.5± 12.8 *vs* 62.5±14.0 years respectively. In-hospital mortality was 5.1%, 3.1% and 2.5% for patients with STEMI, NSTEMI, and UA, respectively. In STEMI patients, thrombolytic therapy was performed in 88 (56.5%) patients and 68 (43.5%) had primary coronary angioplasty (PCI). The predictive value of different clinical variables for in-hospital mortality and cardiac events in the study were: 2.8 for GRACE score >170, 3.1 for DM, 2.2 for SBP >180 mmHg, 1.4 for age >65 years, 1.8 for BMI >28, 1.7 for creatinine >160 μmol/L, 2.1 for HR >90 bpm, 2.2 for positive serum troponin and 2.3 for ST elevation.

**Conclusion:**

Patients with STEMI compared with NSTEMI and UA were of younger age. There was higher in-hospital mortality in STEMI compared with NSTEMI and UA patients. The most significant predictors of death or cardiac events on admission in ACS were DM, GRACE Score >170, systolic hypertension >180 mmHg, positive serum troponin and HR >90 bpm.

## INTRODUCTION

Substantial proportions of patient’s burden of Coronary Artery Disease (CAD) are attributed to major risk factors [1]. Acute Coronary Syndrome (ACS) refers to clinical symptoms that are compatible with acute cardiac ischaemia, with a spectrum ranging from unstable angina (UA) to a non-ST Segment Elevation Myocardial Infarction (NSTEMI) to ST-Segment Elevation Myocardial Infarction (STEMI) [2, 3].

The mechanism of acute ischaemia is largely due to atherosclerosis process and plaque disruption affecting the coronary arteries. Several coronary risk factors may influence this process, including hypercholesterolaemia, hypertension, diabetes and smoking [4, 5]. The major risk factors may affect the endothelium of the blood vessel resulting in endothelial dysfunction which plays a pivotal role in initiating the atherosclerotic process [6, 7]. In case of acute chest pain, a 12 lead electrocardiogram (ECG) distinguishes between various clinical conditions. The ECG should be done in an ambulance if possible [8-10]. ACS is a common manifestation of cardiovascular disease and its recognition with appropriate treatment will influence mortality and morbidity [11, 12].

There is no substantial clinical data in the Kingdom of Bahrain on the pattern of various clinical presentations of ACS and the impact of different risk factors on cardiac events in the first 48 h after hospital admission.

The current study aims to: (1) determine the clinical risk profiles of patients presenting ACS in the Kingdom of Bahrain, (2) describe the clinical cardiac events and in-hospital mortality of these patients, and (3) to evaluate the predictive value of different variables for risk of in-hospital death and cardiac events.

## 2 METHODS AND MATERIALS

This is a prospective, observational, and consecutive study of all patients who were admitted to the Salmaniya Medical Complex (SMC) and the Bahrain Defence Force Hospital (BDF) with a confirmed diagnosis of ACS. Enrolment was over 12 months from the first of January 2012 to the first of January 2013. The study protocol was approved by the Ethics Committee of Salmaniya Medical Complex. A consent form was signed by every patient enrolled in the study.

### 2.1 Study Population

Bahraini patients (n=635), aged ≥18 years with a confirmed diagnosis of ACS according to hospital code of admission were enrolled in the study.

### 2.2 Initial Clinical Evaluation

The primary focus in the early evaluation, within the first 15 min after presentation of patients with possible ACS, is to confirm or exclude ACS as a cause of the symptoms. In this period of time, the confirmation of ACS comes from diagnostic ECG changes [13].

There are 3 types of ACS: STEMI, NSTEMI and UA. The first two are characterized by a typical rise and fall in serum troponin [14]. UA is characterized by myocardial ischaemia without elevated biomarkers and is often a clinical diagnosis based on history and dynamic ECG changes.

All pre-hospital evaluation of patients with ACS were performed by obtaining a pre-hospital 12-lead ECG and continuous ECG. A reperfusion protocol is then initiated after pre-hospital diagnostic or on hospital arrival ECG.

Patients with symptoms of acute myocardial ischaemia had continuous ECG monitoring. Initial 12-lead ECG performed within 10 min of arrival, even if one had been performed in an ambulance, unless that ECG showed ST elevation with chest pain or symptoms that could represent myocardial ischaemia-like shortness of breath, palpitation, syncope or generalized weakness [15].

### 2.3 Data Collection

Clinical data and clinical variables were in accordance with key data elements and definitions of patients with ACS [16].

Data collection included: height, weight, body mass index (BMI), age, gender, marital status, medical history (including CAD risk factors), hypertension, smoking, diabetes mellitus, hyperlipidaemia and medication. The clinical presentation depended on admission, management during hospital stay, medications, reperfusion therapy, and procedures performed in the initial 48 h.

Every patient in the study had undergone a 12 lead ECG on admission which was repeated at the 15 min marker, then every 8 h for the first 24 h, then twice daily until discharge.

The medications administered and clinical cardiac events such as arrhythmia, hypotension and pulmonary oedema and cardiogenic shock were all recorded in the first 24 h. The need for reperfusion therapy of acute STEMI either by thrombolysis or primary angioplasty/stenting was also recorded. The severity of pulmonary oedema was graded as per Killip class [17]

Clinical examinations included monitoring the systolic blood pressure (BP), diastolic BP, heart rate (HR), presence or absence of crackles and rhonchi on chest examination, peripheral pulses and the presence of cranial or neurological deficit.

Blood samples were withdrawn on admission to monitor levels of: troponin I, creatine kinase isoenzyme (CKMB), haemoglobin, white blood cell (WBC) count, random glucose and creatinine. A blood sample of fasting sugar (FS) and fasting lipid (including total cholesterol, High-Density Lipoprotein Cholesterol (HDL-C), measured Low-Density Lipoprotein Cholesterol (LDL-C) and fasting triglyceride (TG)), and the level of glycosylated haemoglobin (HbA_1c_) were also measured.

GRACE score (Global Registry of Acute Coronary Events) using 8 clinical parameters was used for risk assessment and the prediction of adverse outcome in patients with ACS. The GRACE Score (GS) risk assessment was performed at the time of hospital admission using the digital online score calculator (http://www.outcomesmassmed.org/ grace/ acs_risk/acs_risk_content.html).

A GS <100 was regarded as low risk, a score of 100-170 as medium risk and >170 as high risk [18]. The 8 parameters assessed in GS were as follows: elevated cardiac marker, age, Heart Rate (HR), systolic BP, Killip class, ST-segment elevation on 12 lead ECG, serum creatinine level and cardiac arrest at hospital admission.

The cut-off point for cardiac troponin I (cTnI) is 0.01 ng/mL and reference value of measured CKMB for males was ≤7.7 ng/ml and for females ≤4.3 ng/ml [19].

### 2.4 Statistical Analysis

The statistical package of SPSS Version 20.1 was used; baseline patient data and patient characteristics were summarized using counts and percentages. Continuous variables were measured using means, standard deviations, confidence intervals and the percentages of each variable in the study population and then calculated and compared with other relevant variables. Multiple logistic regression analysis was applied to evaluate the predictive value of death and cardiac events in patients with ACS. The variables are age >65 years, body mass index (BMI) >28 kg/m^2^, GS >170, history of DM, systolic hypertension >180 mmHg, level of creatinine >160 μmol/l and HR on admission >90 bpm, cTnI rise and ST segment elevation. A two –sided P value <0.05 was accepted as significant.

## 3 RESULTS

A total of 635 patients with a clinical diagnosis of ACS were enrolled over 12 months. The ACS patients, out of 7,450, formed (8.6%) of patient admissions with acute medical problems in both hospitals. The mean age of ACS patients was 61.3 ±13.2 years (range 18-85); 417 were males (65.6%).

Table (**1**) shows the age categories of patients with ACS as follows. The majority of patients with ACS (75%0) were in the age category of 45-74 years, followed by age <45 years (12.0%) and those over 75 years (13.0%).

### 3.1 Haemodynamic Clinical Condition on Admission

There were 211 (33.2%) patients with systolic BP on admission >140 mmHg, and =140 (22.0%) patients in whom the diastolic BP >100 mmHg, all these patients gave a history of hypertension. There were 331 (52%) patients with a HR >70 bpm.

### 3.2 Clinical Presentation of Patients with ACS

Chest pain in ACS patients was typical cardiac pain radiating to the left arm in 439 (69%), 47 (7.4%) patients had atypical chest pain without radiation, 116 (18%) had shortness of breath with mild chest tightness, 17 (2.6%) had fainting episodes, 11 (1.7%) had a sense of nausea, vomiting and feeling unwell and 5 (0.7%) patients had palpitations.

### 3.3 Cardiac Events Among Patients with ACS

There were 11 (1.7%) patients suffering from cardiac arrest on admission, 7 patients had ventricular tachycardia after admission and 4 patients had ventricular fibrillation that degenerated into asystole outside the hospital. Ten (1.6%) patients had in-hospital supraventricular tachycardia (SVT) and 25 (3.9%) had Atrial Fibrillation (AF). There were 5 patients with a history of AF on admission and AF occurred in 20 patients within the first 24 h.

### 3.4 Heart Failure

Fig. (**1**) shows the patients with Killip Class (KC) of pulmonary oedema on admission. Four hundred and eighty-nine (77%) patients had no pulmonary oedema (Killip class 1), 146 (23%) patients had pulmonary oedema with lung crackles clinically. Out of those 83 (13%) had pulmonary oedema but normal BP >120/80 mmHg (Killip class 2), 50 (8%) had pulmonary oedema and hypotension of <100/70 mmHg (Killip class 3) and 13 (2%) patients had cardiogenic shock (Killip class 4).

### 3.5 ECG Changes on Admission

There were 156 (24.5%) patients with STEMI with cardiac enzyme rise, 158 (25%) patients had no ST elevation but ST depression myocardial infarction (NSTEMI) with cardiac enzyme rise. There were 321 (50.5%) patients with unstable angina (UA) with neither ST elevation nor cardiac enzyme rise. Twenty-eight (4%) patients had left bundle branch block (LBBB) on admission, 8 of them were new and 16 old LBBB. Twelve patients with LBBB had cardiac enzyme rise and 5 patients had old right bundle branch block (RBBB) with no enzyme rise.

### 3.6 Rhythm on 12 Leads ECG on Admission

There were 576 (90.5%) patients in sinus rhythm (SR), atrial fibrillation (AF) in 48 (7.6%) patients, 20 patients had new onset AF and 7 (1.1%) patients had pacing rhythm and 12 (1.9%) patients had a permanent pacemaker.

The initial serum level of cTnI and CKMB in patients with ACS were 3.1±0.3 ng/ml and 45.3±0.6 ng/ml, respectively, while the peak serum level was 18.8±2.6 ng/ml and 72.7± 0.6 ng/ml, respectively. The mean of random blood glucose on admission was 11.7±2.1 mmol/l and the fasting glucose was 8.2 ±1.8 mmol/l. The level of HbA_1c_ was 7.6±1.1% on admission in those with a history of DM. Fasting total cholesterol was 5.2± 1.1 mmol/L, triglyceride was 1.6±0.5 mmol/L, LDL-C was 2.9 ±1.1mmol/L and HDL-C was 1.1± 0.2 mmol/L.

### 3.7 Prior Medication on Admission

There were 325 patients taking aspirin, 77 on clopidogrel, 105 on angiotensin receptor antagonist (ARB), 233 on angiotensin converting enzyme inhibitors (ACEI), 292 on beta-blockers (BB), 244 on statins, 97 on oral isosorbide mononitrate, 119 on a diuretic, 17 on warfarin and 115 on a calcium channel blocker (CCB).

### 3.8 Past Medical History of Other Cardiac Risk Factors

There were 221 (34.8%) patients with history of hyperlipidaemia, 294 (46%) had DM; 25 (4%) with type 1 DM and 269 (42%) with type 2 DM, those with history of hypertension were 311 (49%) and there were 276 (43%) patients with a BMI >28 kg/m^2^ and 185 (29%) with a positive family history of CAD. The risk factors in ACS patients and the control group are compared in (Table **2**).

### 3.9 History of Non-Cardiac Disease

Six patients had a history of transient ischaemic attacks (TIA), 40 (6%) patients had prior stroke, 30 (4.7%) of them with good recovery. There were 23 (3.6%) patients with prior Peripheral Artery Disease (PAD); 2 patients had a history of Deep Venous Thrombosis (DVT).

It was observed that 70 (11.4%) patients with ACS had chronic kidney disease (CKD) with 19 (2.9%) patients on regular haemodialysis and 35 (5.5%) patients among those with CKD had PAD, 40 had hypertension and 29 with DM, 3 patients with cardiac arrest were on regular dialysis and they were hypertensive and diabetic.

### 3.10 History of Cardiac Disease in the Study Population

Sixty-three (10%) patients had Prior Percutaneous Coronary angioplasty (PCI), 32 (5%) patients had prior coronary artery bypass surgery (CABG), 7 (1%) had permanent pacemaker implant, 118 (21%) had old myocardial infarction (MI) and 184 (29%) had history of angina pectoris in the past and 57 (9%) patients had congestive cardiac failure.

### 3.11 Smoking Habits

There were 326 (51.5%) patients, who were non-smokers. Current smokers were 173 (27%) patients: 100 (16%) smoked >20 cigarettes/day, 40 (6%) patients smoked between 10-20 cigarettes/day and 33 (5%) were smoking <10/day. There were 136 (21.5%) patients, who stopped smoking >1 year.

### 3.12 Management of Patients after Admission

*In* patients with STEMI (n=156). Eighty eight patients (56.5%) had thrombolysis, 44 (28%) had primary coronary angioplasty/stenting (PCI) and 24 (15.5%) had salvage PCI, 12 of them were not given thrombolytic therapy due to late admission of more than 12 h after onset of pain, 5 had contraindication of thrombolysis and 9 (1.4%) had failure of thrombolytic therapy. All patients with DM type were administered insulin infusion on admission and 20 with type 2 DM were given insulin due to high glucose (>15 mmol/L) on admission, the rest of the DM patients were given subcutaneous injection according to sliding scale for 48 h after admission.

### 3.13 GS in The Study

Fig. (**2**) summarizes the occurrence of high or low GS in the 3 categories of ACS. For patients with STEMI, 58% of patients had GS of >170, 26% had GS of 100-170 and 16% of <100. For NSTEMI patients 23% had GS >170, 51% of 100-170 and 26% of <100. In patients with UA 8% had GS of >170, 19% of 100-170 and 73% of <100.

### 3.14 Echocardiogram on Previous Admissions to Hospital

Among those who had previous echocardiogram, there were 362 (57%) patients with normal LVEF >45%. LVEF dysfunction of <45% observed in 28 (4.4%) with mild dysfunction of LVEF between >35-≤45%, 68 (10.7%) had moderate dysfunction with %LVEF between >25 - ≤35% and 25 (3.9%) had severely low %LVEF (<25%).

### 3.15 Risk prediction in ACS Patients for Death and Cardiac Events

Multiple logistic regression analysis results of the ACS patients are tabulated in Table (**3**). The most significant variable was DM followed by GS >170 and systolic hypertension on admission of >180 mmHg, troponin rise suggesting myocardial necrosis and HR >90 bpm.

## DISCUSSION

The demographic trends in ACS worldwide are changing due to primary prevention measures and because of acute therapeutic interventions. The incidence of ACS over 1 year in Bahrain was (1/1000), as 635 patients out of 630,000 Bahraini citizens in the Kingdom of Bahrain were admitted. The incidence in this study was lower than that of the CZECH registry where the calculated annual incidence of confirmed ACS was 3248 cases/million population [20]. The low incidence in Bahrain could be because not all patients with ACS were admitted into the two major public hospitals.

In the current study, the mean age of patients who presented with ACS was 61±13 years; that is higher than in developing countries like Saudi Arabia [21]. This difference may be due to the heterogeneous ethnicity and different risk factors. In our study, ACS patients compared with the control group had a prevalence of 46 *vs* 17% for DM which is 2.7 times higher in ACS patients; 52 *vs* 48% for history of hypertension, 46 *vs* 36% for obesity of >28 BMI, 43 *vs* 40% for hyperlipidaemia and 27 *vs* 20% for current smoking.

The history of risk factors in this study is lower than that of ACS patients in the Gulf region as: 66% for hypertension, 55% for hyperlipidaemia, 53% for DM and 23% for current smokers [22]. In one observational study of the healthy Bahraini population, the prevalence of DM was 14.3%, hyperlipidaemia was 40.6%, obesity was 36.3%, hypertension was 48.2% and smoking was 19.9% (National Non-communicable Diseases Risk Factors Survey in 2007) with clearly high rate of hypertension and obesity even in healthy volunteers [23].

The prevalence of male gender (65%) was high in the current study. This finding is in keeping with previous reports where males had a higher rate of ACS *vs* females [16]. In the Gulf RACE study, the prevalence of ACS in male was 74% and in female was 24%; obesity was observed to be at highest level in the middle age groups between (30-49 years) [24].

The incidence of AF in the study was (3.1%) which is lower than that reported by Zimetbaum *et al.*, where AF as a complication of acute myocardial infarcts was 5.5% [25]. Eldar *et al.* reported 9.8% incidence of paroxysmal AF in a consecutive series of acute myocardial infarction [26]. The incidence of ventricular fibrillation (VF) in acute MI has been reported to be 4.7% [27, 28]. Therefore, despite continuous efforts in primary prevention and population education, it is the extent of myocardial necrosis and the degree of Left Ventricular (LV) dysfunction that determines arrhythmia risk following STEMI. Sustained, monomorphic (ventricular tachycardia) VT usually develops in patients with more extensive MI who also have lower LV Ejection Fraction (LVEF) [29]. In the current study, 1.7% of patients developed VT on admission and VF of 0.06%, which is lower than reported in the literature [30]. This could be due to the fact that majority of patients with VF do not ever come to the hospital and die before admission.

Thirteen % of patients had pulmonary oedema with no hypotension, while 8% had hypotension and only2 percentage had cardiogenic shock. In previous reports, cardiogenic shock occurred among patients with STEMI at a rate of 7.5% [31] and in 2.5% of patients with NSTEMI and 4.2% of patients with STEMI [32].

The history of hypertension in ACS patients at 52% was higher than a previous report (INTERHEART) where 31% of patients with AMI had antecedent hypertension [33]. A study in the USA showed hypertension was an independent factor for major short- and long-term cardiac adverse outcome [34]. Pulmonary oedema with normal BP on admission was detected in 23% of patients studied, 13% had pulmonary oedema with hypotension but only 2% had cardiogenic shock.

Among those with a previous echocardiogram (n=483), an LVEF of <25% was detected in 3.9% and 10.7% had an LVEF of 25-35%.

The majority of patients admitted with heart failure have CAD, which independently has an adverse impact on prognosis. The initial in-hospital and after-discharge management of heart failure may be dependent on the clinical presentation. Patients with ACS complicated by heart failure and the presence of ischaemia or stunned/hibernating myocardium should be assessed for optimal management as per recommendations [35, 36].

Fifty eight percent of patients with STEMI had a high GS of >170 with higher in hospital mortality of 5.1%, while 73% of patients with UA had a low GS <100 with a hospital mortality of 2.5%. This finding is in keeping with a previous report where the GS as predictor of in hospital mortality provided a superior discrimination in myocardial infarction patients compared with the TIMI (Thrombolysis In Myocardial Infarction) score [37].

Patients with STEMI had a high percentage of elevated GS with greater in-hospital death of 5.1%; a lower GS in UA patients was associated with less mortality (2.5%). This finding is in keeping with previous report where lower GS associated with lower mortality [38].

In the current study, 50% of patients were noted to have tachycardia on admission with a HR >70 bpm. In a previous report, an increased HR in ACS was associated with adverse outcomes after AMI, including stroke, heart failure and cardiovascular death [39].

Multiple logistic regression analysis in this study showed that DM and systolic hypertension on admission, with the rise of troponin and high GS >170, were the most significant predictors of in-hospital death and cardiac events in ACS. Therefore, it is recommended that all patients with NSTEMI or UA should undergo risk stratification, particularly those with ST-depression on the ECG or elevated cardiac biomarkers. It is preferable to use GS tools, as they are derived from large populations and have been validated in the gulf region [39].

For patients with known CAD, the additional presence of PAD considerably worsens prognosis [40, 41]. Furthermore, the observed high incidence of CKD in the study at 11% is in keeping with other reports where patients with CKD had a higher incidence of CAD >50% in unselected CKD patients with an increment of mortality rate after AMI [42].

## 5 LIMITATIONS

The registry may be limited by selection bias. The exclusion of high-risk patients of being enrolled due to refusal or inability to sign the consent form on admission. This bias may affect the clinical outcomes in the study.

So in order to reduce this bias, a logbook was introduced and completed for all patients who were not enrolled. The logbook showed that 10 patients refused to enter in the study. Among them 3 had UA, 4 had NSTEMI and 3 had STEMI with 2 that opted for thrombolytic therapy and 1 that had coronary angioplasty stenting.

Furthermore, this study was conducted among Bahraini patients only; non-Bahraini patients were not enrolled. However, the difference in ethnicity, social and demographic aspects of non-Bahraini patients adds strength to the study population homogeneity.

## CONCLUSION

Patients with STEMI compared with NSTEMI and UA were of younger age. There was higher in-hospital mortality in STEMI compared with NSTEMI and UA. The most significant predictors of death or cardiac events on admission were DM, GS >170, systolic hypertension >180 mmHg, positive serum troponin and HR >90 bpm.

## Figures and Tables

**Fig. (1):**
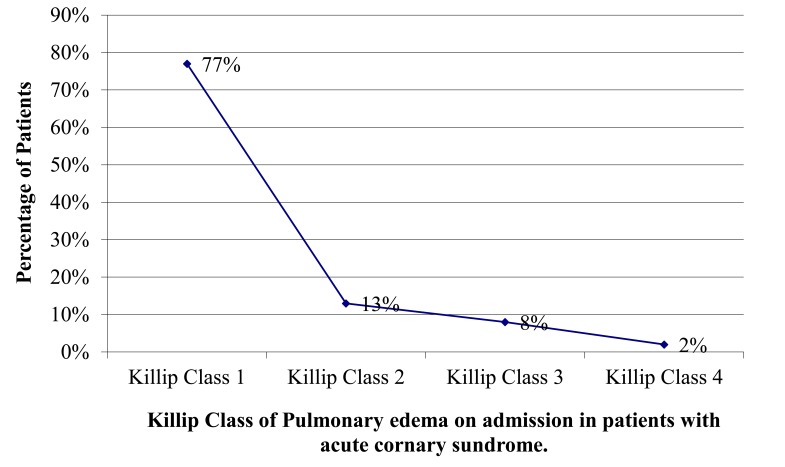
Patients with ACS (n=635), percentage of pulmonary edema (PO) on admission. class (KC),KC1:no edema, with normal blood pressure, KC3: edema and hypotension, KC4: cardiogenic shock.

**Fig. (2):**
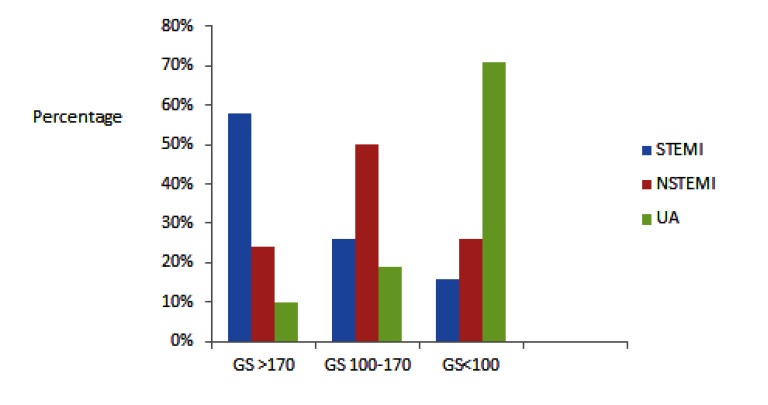
Grace Score (GS) in patients with ACS with STEMI n= 156, STEMI, n= 158, UA, n= 321

**Table 1:** Distribution of age categories among patients with ACS in Bahrain over 12 months.

**Age Category (Years)**	**n (%)**
<45	76 (12.0%)
45-54	159 (25.0%)
55-74	317 (49.9%)
≥75	83 (13.1%)

Abbreviation: ACS: acute coronary syndrome.

**Table 2:** Percentage of coronary risk factors in patients with ACS and those without matched for age and gender.

	ACS Patients (n=635)	Non ACS Patients (n=635)
Hyperlipidaemia	43%	34%
Diabetes mellitus	46%	17%
Hypertension	52%	48%
Obesity with BMI >28 kg/m^2^	46%	36%
Family history of coronary artery disease	29%	20%
Smoking	27%	20%

Abbreviation: ACS: Acute coronary Syndrome, BMI: body mass index

**Table 3:** The results of multiple logistic regression analysis in patients with ACS in term of predictive value for mortality and cardiac events.

Variable	Odds Ratio	Confidence Interval (CI)	p
Age >65 years	2.3	1-1.8	<0.05
BMI >28 Kg/m^2^	1.8	1.4-2.2	<0.01
Diabetes mellitus	3.1	2.5-3.7	<0.01
Systolic hypertension	2.2	1.8-2.6	<0.01
GRACE score	2.5	2-3	<0.01
Creatinine >160 μmol/L	1.7	1-2.4	<0.05
Troponin rise	2.4	1.8-3	<0.01
Heart rate >90 bpm	2.1	1.6-2.7	<0.01
ST segment elevation or depression	2.3	1.8-2.8	<0.05

Abbreviation: BMI (Body mass index), GRACE: Global Registry of acute coronary Events, ACS: acute coronary syndrome.
